# Use of apical suction to facilitate extra-anatomic bypass for recurrent coarctation: a case report

**DOI:** 10.1186/1749-8090-1-9

**Published:** 2006-03-28

**Authors:** Manoj Kuduvalli, Colin Monaghan, Brian M Fabri

**Affiliations:** 1 Department of Cardiothoracic Surgery, The Cardiothoracic Centre, Liverpool, UK; 2 Specialist Registrar, Cardiothoracic Surgery, The Cardiothoracic Centre NHS Trust, Thomas Drive, Liverpool, L14 3PE, UK

## Abstract

The use of apical suction devices has been well described for maintaining satisfactory haemodynamics during off-pump surgical coronary revascularization. Its expanded use has been described in a few other situations. We describe here a case of recurrent coarctation where an extra-anatomic ascending to descending thoracic aorta bypass graft was constructed using cardiopulmonary bypass without arresting the heart, and access and exposure were facilitated by the use of an apical suction device.

## Case report

**Figure 1:**
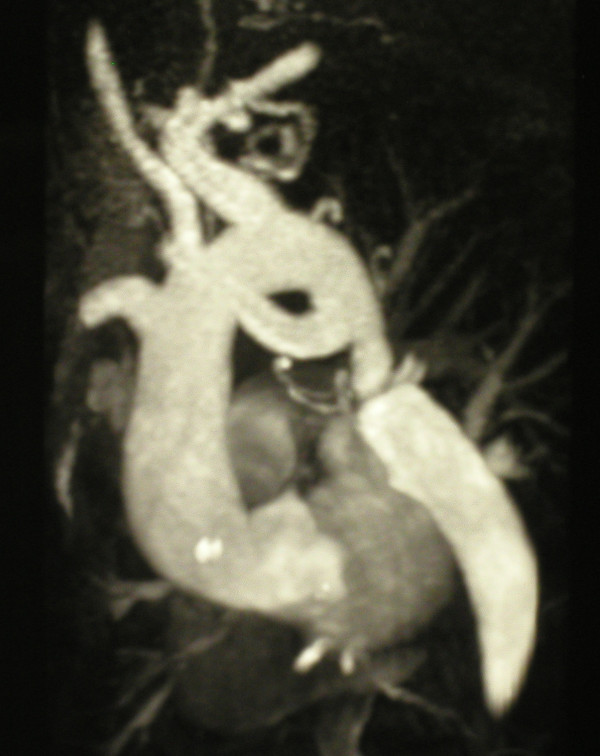
MRI scan showing the re-coarctation.

A 49 year old gentleman presented to cardiology with lower limb claudication pain and breathlessness of three years duration. Clinical examination revealed upper limb hypertension, with similar blood pressures in both arms (180/100 mm Hg). His past history included repair of coarctation of aorta about 30 years ago. The medical records and operative details from the previous operation were unavailable. The operation had been performed through a left thoracotomy. An MRI scan revealed a 2 cm long narrowing of the aorta just distal to the origin of an aberrant right subclavian artery, which was the last of four branches from the aortic arch (Fig. 1). The origins of the arch vessels did not show any sign of narrowing. The aortic root and ascending aorta were 3.5 cm in diameter, and the arch was of normal calibre. The diameter in the region of the stenosis was 1.4 cm with an additional web-like stenotic lesion at the distal end of the stenotic segment. There was evidence of calcification, possibly of an interposition tube graft which had been used at the time of the first operation. The descending thoracic aorta was of normal calibre.

In view of his symptomatic status, a re-intervention was considered appropriate. In view of his previous surgery, and especially the fact that the area of re-coarctation appeared to be calcified, it was decided to approach the aorta via a median sternotomy and construct an extra-anatomic ascending to descending thoracic aorta bypass graft. Cardiopulmonary bypass would be necessary to lift the heart out of the way to gain access to the descending thoracic aorta just above the diaphragm. We planned to use an apical suction device to keep the empty beating heart elevated.

**Figure 2:**
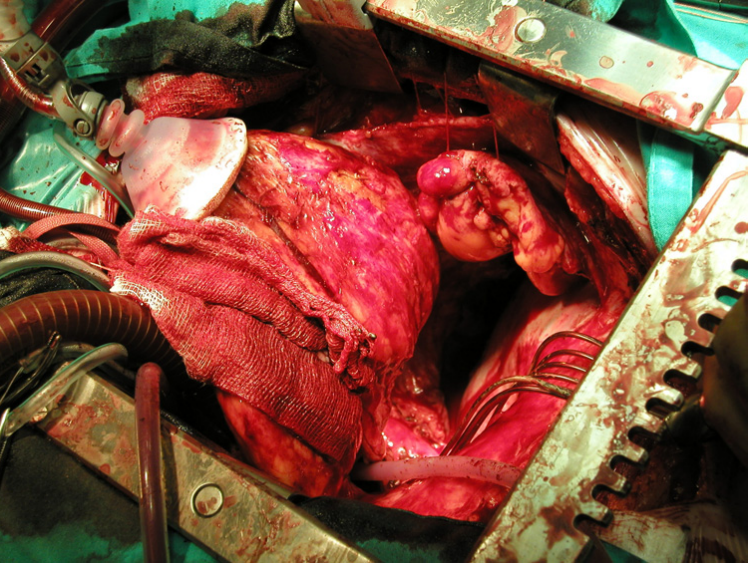
Exposure of the descending thoracic aorta with the apical suction device retracting the heart.

The sternotomy was completed uneventfully. The pericardial cavity was obliterated with dense adhesions. This was rather surprising since we had anticipated that the previous procedure would have been extra-pericardial. However, further dissection revealed a large hole in the pericardial sac with the left lung directly adherent to the heart. The adhesions were released, some of them after establishing cardiopulmonary bypass using ascending aortic cannulation for inflow and bicaval cannulation (to maintain adequate venous drainage even after lifting up the heart) for venous outflow. Once the apex and the posterior surface of the heart were free of adhesions, an apical suction device (URCHIN™ Heart Positioner, Medtronic Inc., Minneapolis MN55432-5604 USA) was placed in position and the beating heart was lifted superiorly. This allowed further dissection in the posterior pericardium and allowing freeing up of adhesions between the left lung and the descending thoracic aorta, and allowed visualization of and access to the descending thoracic aorta just above the diaphragm in spite of a deep thoracic cavity (Fig. 2). Proximal and distal cross clamps were applied isolating a 4 cm length of aorta. A longitudinal incision was made in this segment and an 18 mm Haemashield Platinum™ Woven Double Velour Vascular tube graft (Boston Scientific Corporation, Natick, MA 01760-1537) was anastomosed in an end to side manner using continuous 3-0 polypropylene sutures. The clamps were released, the distal one first, the aorta was de-aired and the anastomosis was checked. The graft was then routed to the right of the inferior vena cava and brought up alongside the right atrium to the ascending aorta. The apical suction device was released and the heart was replaced in the pericardial sac. The length of the tube graft was estimated after filling up the heart. A side biting clamp was applied to the ascending aorta and the proximal anastomosis of the tube graft was constructed to a longitudinal arteriotomy using 3-0 polypropylene sutures. The clamp was released, the graft was de-aired, and the anastomosis checked. The patient was weaned off cardiopulmonary bypass with no inotropic support. Haemostasis was ensured and the chest was closed in the routine manner leaving two drains in the left pleural space, one drain in the pericardial sac and one in the mediastinum.

**Figure 3:**
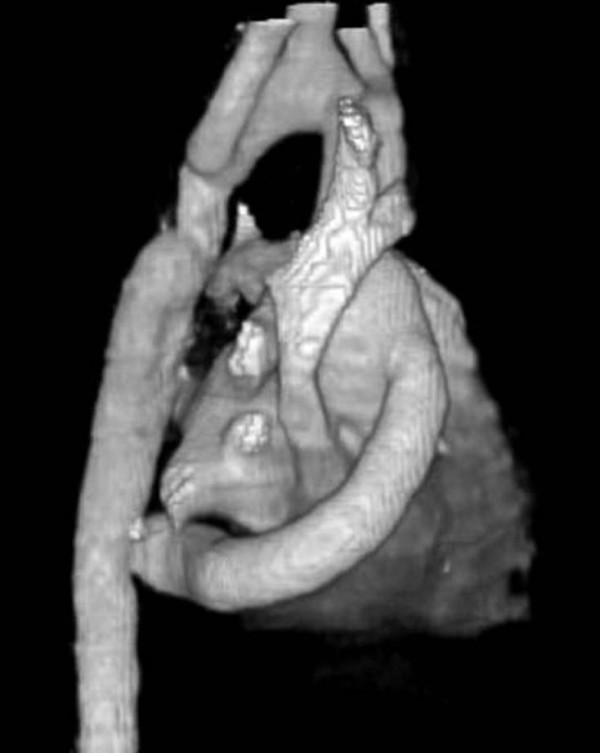
3-D reconstruction of postoperative CT scan showing the extra-anatomic bypass graft.

The patient was extubated eight hours after arrival in the intensive care unit. His drains were removed the next morning. He made an uneventful recovery thereafter except for needing some respiratory support with non-invasive continuous positive airway pressure for treating basal atelectasis. A CT scan was done prior to his discharge from hospital on the tenth postoperative day. Figure 3 shows an oblique 3-D reconstructed view from the CT scan demonstrating the locations of the proximal and distal anastomoses, and the lie of the graft. The patient was reviewed in outpatients six weeks after his discharge. His claudication pain had disappeared completely. His upper limb blood pressure was 120/60 mm Hg on a reduced amount of medication.

## Comment

The use of apical suction devices for cardiac positioning in off pump coronary artery surgery, and its ability to maintain good beating heart dynamics has been described [1,2]. Apart from being used in off-pump coronary artery bypass grafting, the apical suction device has been described to have expanded uses in a variety of scenarios. It has been described for use in pericardectomies, during lysis of adhesions in redo coronary surgery, for securing epicardial haemostasis in penetrating cardiac trauma, for securing haemostasis during re-exploration after cardiac surgery and for facilitating epicardial microwave ablation [3].

Its use has also been described in a case of recurrent coarctation in which an ascending-to-abdominal aorta bypass graft was successfully facilitated by the use of an apical suction device [4]. The operation was done without the use of cardiopulmonary bypass.

In the case described in our report, the use of cardiopulmonary bypass was necessary because the heart would probably not have tolerated the amount of elevation which would have been required to expose the descending thoracic aorta just above the diaphragm, which was our target area for constructing the anastomosis. However, our rationale for use of the apical suction device on the empty beating heart was: 1) to avoid cross clamping the heart for a prolonged period of time for an extra-cardiac operation 2) to make the elevation and retraction of the empty beating heart technically easier, and less traumatic on the epicardium and myocardium compared to retracting and elevating with the use of the assistant's hand. We could accomplish both these objectives safely and successfully, with adequate exposure to clamp the descending thoracic aorta and perform the anastomosis.

This report describes another expanded use for the apical suction device.
